# Applying WHO COVID-19 workforce estimate tools remotely in an African context: a case report from Mali and Kenya

**DOI:** 10.1186/s12960-021-00653-5

**Published:** 2022-01-28

**Authors:** Pamela A. McQuide, Amy Finnegan, Katherine M. Terry, Andrew Brown, Cheick Oumar Toure, Jeanne Tessougue, Ibrahim Cisse, Mathew Kariuki Thuku, Janet Muriuki, Mary Ochola, Julius Ogato, Etienne Coulibaly, Toure Djeneba Togora

**Affiliations:** 1 IntraHealth International, Chapel Hill, NC USA; 2 IntraHealth International, Bamako, Mali; 3 IntraHealth International, Nairobi, Kenya; 4 Coast General Teaching and Referral Hospital, County Government of Mombasa, Mombasa, Kenya; 5 Ministry of Health, Nairobi, Kenya; 6 Ministry of Health, Bamako, Mali

**Keywords:** COVID-19, Health workforce, Kenya, Mali, Workload modelling, WISN

## Abstract

**Background:**

The COVID-19 pandemic has increased the burden on health systems, particularly in low- and middle-income countries, where health systems already struggle. To meet health workforce planning needs during the pandemic, IntraHealth International used two tools created by the World Health Organization (WHO) Regional Office for Europe. The Health Workforce Estimator (HWFE) allows the estimation of the quantity of health workers needed to treat patients during a surge, and the Adaptt Surge Planning Support Tool helps to predict the timing of a surge in cases and the number of health workers and beds needed for predicted caseload. These tools were adapted to fit the African context in a rapid implementation over 5 weeks in one region in Mali and one region in Kenya with the objective to test the feasibility of adapting these tools, which use a Workload Indicators of Staffing Need (WISN)-inspired human resources management methodology, to obtain daily and surge projections of COVID-19 human resources for health needs.

**Case presentation:**

Using a remote team in the US and in-country teams in Mali and Kenya, IntraHealth enacted a phased plan to gather stakeholder support, collect data related to health systems and COVID-19 cases, populate data into the tools, verify modeled results with results on the ground, enact policy measures to meet projected needs, and conduct national training workshops for the ministries of health.

**Conclusions:**

This phased implementation in Mali and Kenya demonstrated that the WISN approach applied to the Health Workforce Estimator and Adaptt tools can be readily adapted to the local context for African countries to rapidly estimate the number of health workers and beds needed to respond to the predicted COVID-19 pandemic caseload. The results may also be used to give a proxy estimate for needed health supplies—e.g., oxygen, medications, and ventilators. Challenges included accurate and timely data collection and updating data. The success of the pilot can be attributed to the adapted WHO tools, the team composition in both countries, access to human resources data, and early support of the ministries of health, with the expectation that this methodology can be applied to other country contexts.

## Background

The World Health Organization (WHO) declared Coronavirus Disease 2019 (COVID-19) a pandemic on March 11, 2020, impacting countries across the world [1]. Africa had its first recorded case in Egypt on February 14, 2020, and the first case in sub-Saharan Africa on February 27, 2020 in Nigeria [2]. Since the pandemic impacted the African continent more than 1 month after the first reported cases in China, many countries in Africa were initially successful in prolonging the containment phase [2]. Nonetheless, the pandemic has burdened health systems and health workers in low- and middle-income countries [3].

To meet the needs of planning health workforce requirements during the pandemic, the WHO Regional Office for Europe developed two Excel-based tools: the Adaptt Surge Planning Support Tool and the Health Workforce Estimator (HWFE). These tools use a similar methodology and concepts as the Workload Indicators of Staffing Need (WISN) methodology to estimate hours per patient day to care for COVID-19 patients by level of severity, staff needed by cadre and levels of severity to care for COVID-19 patients, and workload pressure to know which cadres or hospitals need the most support and to obtain daily and surge projections of COVID-19 human resources for health (HRH) needs. These underlying WISN principles have been available for the last 20 years [4]. The Adaptt tool combines epidemiological modelling and workload estimates and bed needs for anticipated COVID-19 surges, with reference to local factors such as mitigation and suppression activities and potential number of people exposed to a COVID-19-positive individual [5]. The data show both the dates that predicted shortages will occur and the number of beds and health workers per cadre that will be required to meet predicted needs. The HWFE uses local health workload data, country-level activity standards, and locally available health workers to estimate the number of health workers per cadre needed, with consideration to the number of mild, moderate, severe, and critical patients per day as well as accounting for the number of health workers potentially out of work related to acquiring COVID-19 themselves. Combining these data with existing country health workforce data allows for the visualization of predicted workforce needs and gaps so that countries can anticipate and plan for projected shortages [6].

When the pandemic began to reach African countries, a need for planning the COVID-19 health workforce response was identified. The objective was to gather appropriate data and apply tools that had been already validated in Europe to meet the needs of African countries to estimate health worker and bed requirements for COVID-19. Working remotely, a team from IntraHealth International tailored the Adaptt and HWFE tools to fit the African context in a phased implementation plan over the span of 5 weeks in one region in Mali and one region in Kenya. Adaptations to the original tools included measures such as restructuring work cadres, removing medical interventions not used in Mali and Kenya such as extracorporeal membrane oxygenators (ECMO) and continuous renal replacement therapies, and redefining activity standards to align with local norms. The response needed to be implemented rapidly because of the urgent planning needs imposed by the pandemic. IntraHealth provided remote technical assistance from the US, with support from the WHO Adaptt and HWFE tool developers and consultation with regional WHO staff. IntraHealth funded the work.

## Case presentation

The decision to implement the rapid COVID-19 workforce assessment pilots in Mali and Kenya was based on the rising numbers of COVID-19 cases in each country and the presence of local IntraHealth offices with experience in HRH and in implementing the WISN method, and established human resources information systems and relationships with the ministries of health and WHO country representatives.

Kenya reported its first case on March 13, 2020, and as of April 20, 2020 had reported a cumulative total of 281 confirmed positive cases [7]. Since the beginning of the outbreak and throughout the period of implementing our phased implementation plan in Kenya, the cumulative total of confirmed positive cases increased to 70,804, with 1287 deaths, and an estimated community spread transmission rate of 97% as of November 16, 2020 [8]. The areas with the highest concentration of positive cases continued to be in Mombasa and Nairobi counties, although the virus had spread to all 47 counties by November 16, 2020 [8]. Mombasa County was chosen as the initial county for this pilot, because it was one of the epicenters in Kenya. In particular, the Coast General Teaching and Referral Hospital (CGTRH) was the primary focus, because it was the main COVID-19 treatment center in Mombasa County and the larger Coast region. Similar assessments were done in Nakuru County at the Nakuru County Referral Hospital COVID-19 Isolation Centre and the Naivasha Sub-County Hospital COVID-19 Isolation Centre.

Mali reported its first confirmed positive case on March 25, 2020, and by April 20, 2020 had reported 246 total cumulative cases and 14 deaths [9]. As the epidemic progressed, the reported total cumulative cases rose to 1,961 with 66 deaths by June 22, 2020 [10]. Bamako was selected for the focus of this pilot, because it was the epicenter in Mali for COVID-19 at the inception. The assessments initially focused on three hospitals and later expanded to a total of eight hospitals in Bamako.

The phased implementation plan for the COVID-19 workforce assessments in Mali and Kenya was completed in seven steps outlined as follows:

1. Gather stakeholder support
2. Assemble the implementation teams
3. Select a region and adapt the tools to the local context
4. Collect data
5. Populate the Adaptt and HWFE tools
6. Develop policy measures and apply the results
7. Hold a national workshop to teach local stakeholders how to use the tools.

1. Gather stakeholder support
  The phased implementation plan represented a collaboration among WHO, WHO Regional Office for Europe, WHO Regional Office for Africa (AFRO), WHO country representatives in Mali and Kenya, and the national and regional pandemic response teams in the Ministry of Health (MOH) of Kenya and Mali. These stakeholders remained engaged throughout this phased implementation plan, including final presentations at the conclusion of the initial assessments and later during further scaling of the analysis nationwide.
2. Assemble the implementation teams
  The roles of the in-country implementing teams mirrored those of the remote team at IntraHealth’s home office. These included a project manager/team leader, epidemiologist/data scientist, health workforce/human resources expert, health systems expert, country leader, and a representative from the WHO.
3. Select a region and adapt the tools to the local context
  Country teams decided to begin with one region before scaling to the entire country, choosing the regions with the highest concentration of known COVID-19 cases. The teams then decided which staff categories to include in the health workforce assessment; this varied in each country as different cadres were involved in COVID-19 patient treatment.
  WHO/Europe first introduced the Adaptt and HWFE tools to IntraHealth as they were completing the development of the tools to address COVID-19 needs in Europe in April 2020. The country teams identified the types of specialists and procedures that were not available in the African context and the IntraHealth team adjusted the tools to account for the reality on the ground. In both Kenya and Mali, this included deleting interventions relating to ECMO and renal replacement therapy, which were not available in local hospitals, and removing some of the medical specialist cadres identified in the tools that were not available in these countries. Other adaptations included incorporating hospitalization data for mild cases, since both countries initially hospitalized all cases of COVID-19 to prolong the containment phase and reduce spread of the virus. The clinical expert’s in-country also validated the workload professional activities and activity standards provided in the tools and adapted them to reflect the local context. The professional activities and activity standards indicate how much time is provided by any given cadre by level of severity for specific activities for COVID-19 patient care.
4. Collect data
  Data collection sheets were developed to align with the data entry requirements for the modified Adaptt and HWFE tools, which included specific occupational titles of health workers treating COVID-19 patients, professional activities and activity standards to treat COVID patients, facilities treating COVID with number of beds available for COVID at each facility by severity level, and number of available ventilators by facility. Members of the implementing team in-country gathered the data by accessing the human resources information system (iHRIS) for staffing availability and through direct contact with the facilities targeted. As the data were gathered, it became important to document assumptions made and regularly validate them against actual data to reflect the reality on the ground.
5. Populate the Adaptt and HWFE tools
  The data scientist used the data collection sheets to populate the Adaptt and HWFE tools in a shared drive that the entire team could access and edit in real time. A decision was taken to have one person in charge of inputting data into the tools to ensure that the data remained consistent. The tools were shared with implementing team members during twice weekly meetings so that the team could validate the assumptions with what was happening on the ground.
  The team employed a basic SIR (Susceptible, Infected and Recovered) model embedded in the Adaptt tool to predict the trajectory of the COVID-19 pandemic in Kenya and Mali. At each bi-weekly meeting, the team adjusted the parameters of the model to account for number of contacts and transmission probabilities in each locale to reflect enacted mitigation and suppression measures. Estimates were validated by comparing the cumulative daily cases reported by the national government in each country’s daily situation report to the cumulative cases predicted by the model. As not all cases of COVID-19 were detected, it was important to align the slope of the line as closely as possible to estimate the number of underreported cases. Anecdotally, validation of tool results occurred. For example, the Adaptt tool predicted a bed shortage in Bamako that aligned with an actual bed shortage reported in the local media over the same period.
6. Develop policy measures and apply the results
  After validating the tools, the team’s in-country used the results in collaboration with the MOH and the WHO to enact policy changes and adapt them to the reality of the epidemic. These policy changes included measures such as initiating social distancing policies; hospitalizing all positive cases, including mild and asymptomatic patients; and increasing the number of available beds and health workers in anticipation of the surge. These changes occurred throughout the implementation of the tools based on local context.
7. Hold a national workshop to teach local stakeholders how to use the tools

A 2-day national workshop was conducted remotely to train 30 Kenyan national and county level experts to use the Adaptt and HWFE tools. Representatives included National COVID-19 HRH Committee members, MOH/HRH Unit, Kenya Health HR Advisory Council, health records information officers, epidemiologists, and HRH officers. Hands-on training covered collecting the data and populating the tools to plan for the surge as well as the type of results the tools produce and how to apply the results. Representatives populated the tools with data from Mombasa to familiarize themselves with the tools. In Mali, the WHO funded a similar national training as the one conducted in Kenya. The IntraHealth team conducted a series of remote coaching sessions in both countries to orient the local team members to the tools and build their skills in using them.

## Results and outcomes

**Fig. 1:**
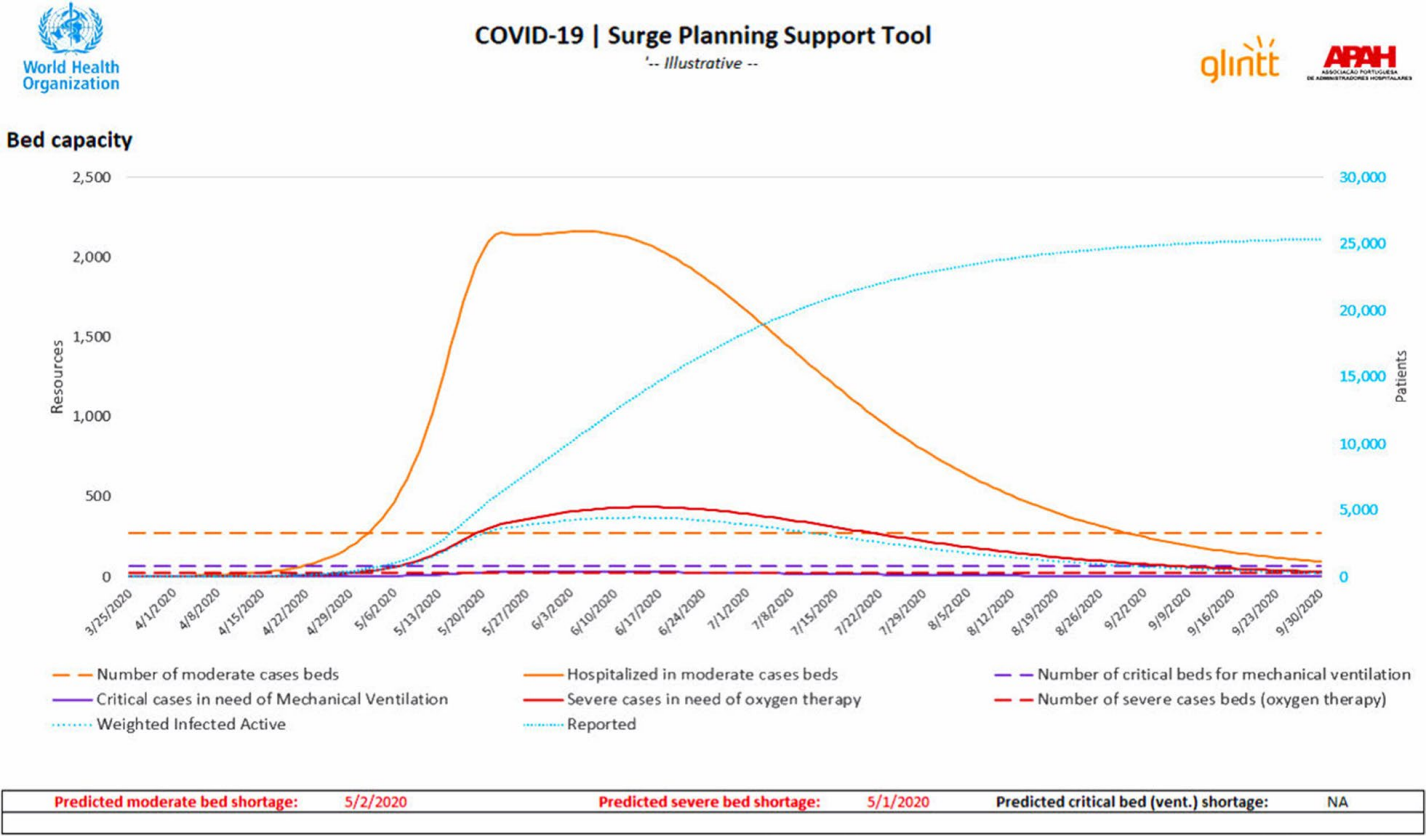
Adaptt tool bed capacity results dashboard indicates when the surge in cases could be expected and the bed requirements by severity level. Results are simulated

**Fig. 2:**
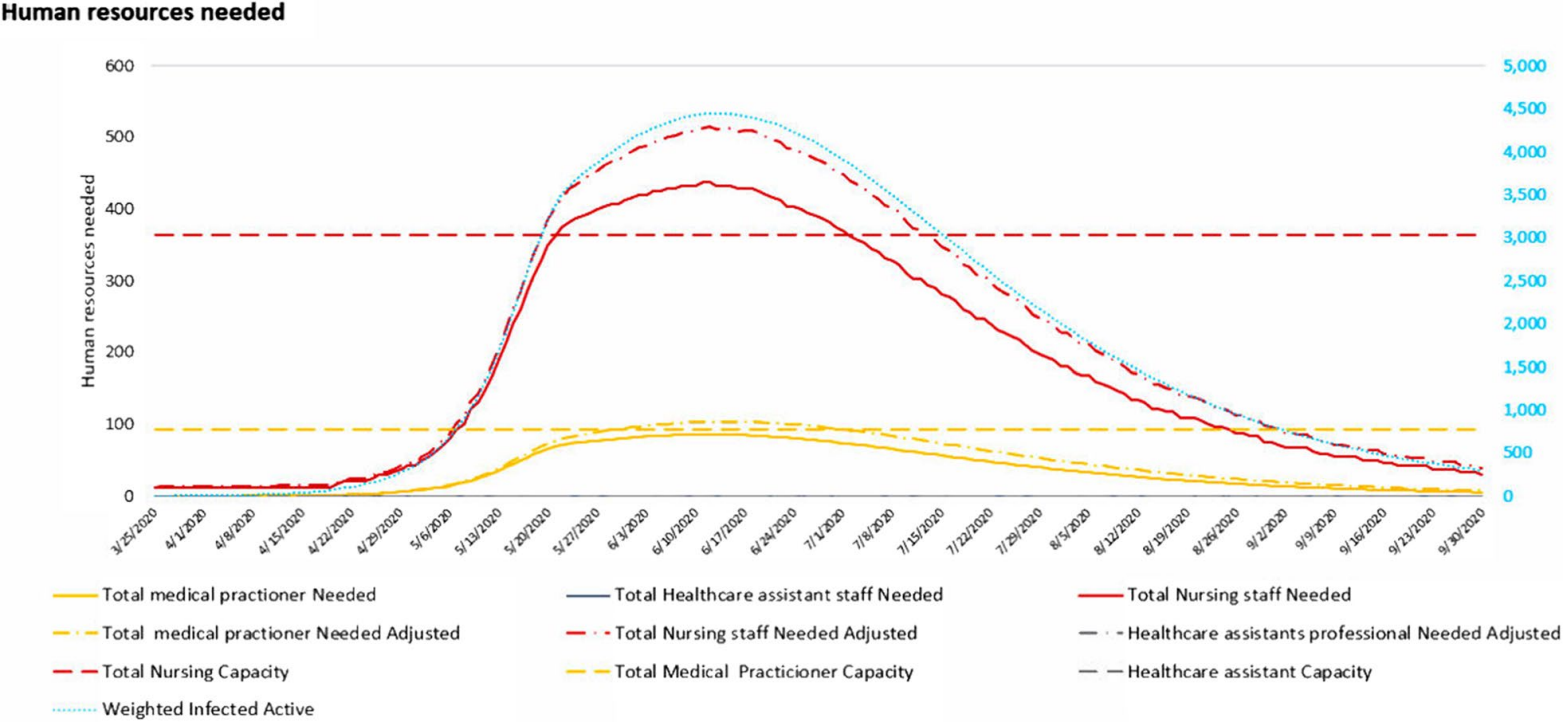
Adaptt tool human resources dashboard indicates when human resources are needed by type based on the estimated cases by severity level. Results are simulated

**Fig. 3:**
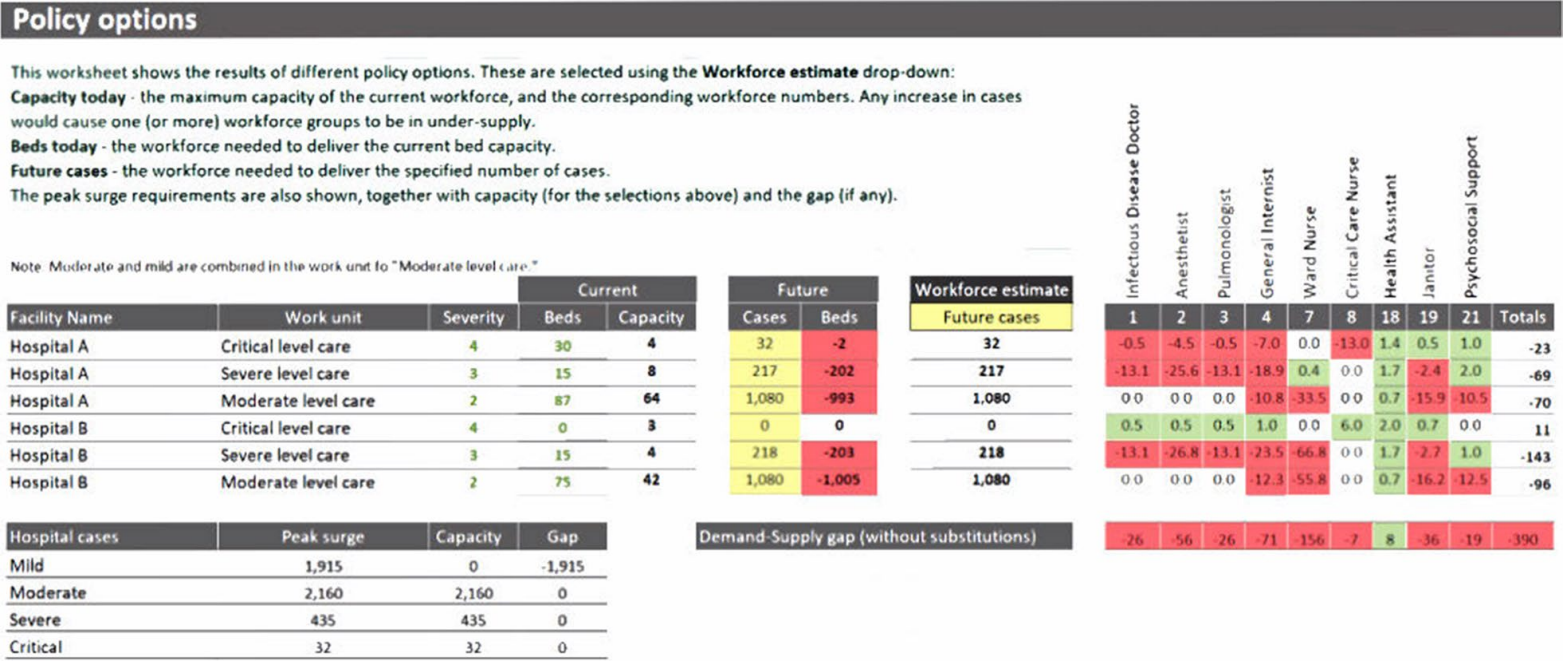
Health Workforce Estimator (HWFE) tool applies the WISN approach to caring for COVID patients and calculates the gap or excess in staff by cadre for the number of expected cases from the Adaptt tool. Results are simulated based on the peak of the surge

The completed Adaptt and HWFE tools yielded dashboards, graphics, and data that provided important real-time information about the anticipated surge of the pandemic and the expected health worker and bed gaps in each of the regions, where the phased implementation plan was conducted. The focus of the Adaptt tool is on forecasting pandemic surge and health worker shortages, while the HWFE is a micro-planning tool that allows hospital managers to reallocate staff to fill shortages identified and plan for the full-time equivalents needed in advance of the surge. The key output of the Adaptt tool was to compare the epidemic surge in cases to the threshold of available staff and beds in graphic form. This simple visual prompted discussions about how to prepare for the anticipated shortages. The epidemiological model results from the Adaptt tool were easily exported to the HWFE tool to estimate the needed staff and beds on any day of the pandemic. Sample graphics using simulated data are provided in Fig. 1 (bed needs), Fig. 2 (human resources needs by cadre), and Fig. 3 (WISN dashboard for the estimated surge in cases) to show the type of results that were available to each country’s MOH to assist in guiding COVID-19 policy decisions.

The results from the tools prompted several adjustments in the COVID-19 response in both Mali and Kenya to meet the needs of the anticipated surge and address workforce gaps expected due to an increase in case numbers.

Kenya is using combined data from this activity to adjust its COVID-19 response. Applying the tools in CGTRH and Mombasa County demonstrated a shortfall of beds and necessary human resources for COVID-19. As a result of the analysis, CGTRH increased the number of beds in the intensive care unit (ICU) to 19, added 100 contingency beds at the hospital, and allocated 300 beds for mild and moderate cases at hotels and universities, with plans to increase to 500 beds. As a further response to the pilot, Kenya’s central MOH and county governments requested a training of trainers on both tools. The tools were scaled up for implementation in Nakuru County after the training in July 2020 and correctly predicted the surge that then occurred in October 2020. The analysis in Nakuru identified a shortage of 150 hospital beds for mild to moderate patients, 89 beds for severe COVID-19 cases in need of oxygen therapy, and an additional 14 beds for critical cases requiring the ICU. The assessments enhanced MOH advocacy across all counties for utilization of a COVID-19 HRH protocol that proposed staff by cadre for the COVID-19 centers to alleviate staff shortages.

In Mali, use of the tools in Bamako demonstrated a shortfall of beds and necessary HRH cadres for COVID-19. In response, Mali’s MOH took prompt corrective actions including:

- Increasing the number of COVID beds in the main teaching hospital in Bamako by 45 to 264.
- Mobilizing additional health workers in Bamako including increasing workers providing psychosocial support from 1 to 10; adding 25 hygiene and cleaning staff; internally reassigning nurses from other hospital wards that were closed due to COVID-19 to increase capacity by 15%; and mobilizing medical residents and student doctors/nurses.
- Paying incentives to all health workers involved in COVID-19 management.
- Increasing COVID-19 treatment facilities in Bamako to include two private hospitals in anticipation of the surge.
- Ramping up HR planning to meet the anticipated surge in Timbuktu, Koulikoro, and Kayes.
- Mobilizing HR clusters in all regions of Mali to conduct surge analysis using the modified tools.

## Conclusions and discussion

### Challenges and lessons learned

The main challenges faced by the implementation teams centered on access to accurate and timely data, as well as region-specific challenges. It was not possible to systematically obtain daily hospital data that presented cases by severity to compare against model predictions. This pilot demonstrated the importance of having such data disaggregated by mild, moderate, and severe patients, which is now being collected by COVID-19 teams. Due to the COVID-19, emergency staff at hospitals were not consistently available to give the in-country team updates. It was also challenging to obtain the number of staff assigned specifically to COVID-19 patients as the situation on the ground was fluid. Therefore, estimates needed to be made based on the total number of beds allocated and use a ratio of staff to estimate the number of health workers by cadre assigned to COVID-19 patients. On several days each week the team adapted the total number of cases nationally and the ratio of patients by region to estimate the new cases per day until actual data from the regional teams at each of the hospitals could be obtained. In Kenya’s devolved health system, it was necessary to work with the county officials equally with the central MOH, since each level of government is independent. Conversely, in Mali, the central MOH led the information sharing at the regional level, which was later expanded to all regions and overseen by the national team.

### Data collection challenges

Challenges with data collection included obtaining accurate staffing data, case data at the city and hospital level, and data on case distribution across COVID-19 severity categories and updating data as the situation changed. These challenges occurred for several reasons. First, facilities were responding to the pandemic, which left little free time to devote to data collection. Second, the process of obtaining national approval to gather local data took several days or weeks, which caused delays in mounting a rapid response. Third, some regions also relied on traditional paper charting, which made it difficult to translate data to an online database. Fourth, since the implementation was done remotely, the staff for our in-country team in Nairobi had to rely on very busy county and hospital staff to give daily updates on the number of cases and changes in mitigating factors. This experience was similar in Mali.

In future phased implementation plans, it is recommended that countries investigate what types of clinical, bed, and HRH data are available before initiating the activity so that they can be prepared with a solution for gathering the required data.

### Staffing data

In both countries, it was possible to obtain the total number of health workers by cadre per hospital from the HRIS, which use the iHRIS software supported by IntraHealth. In Mali the number of staff dedicated to COVID-19 per facility by cadre was also available, but it was not possible to obtain the distribution of staff by severity of COVID cases. In Kenya, the number of staff assigned to COVID-19 cases was unavailable at the beginning of the study. Initially a simple ratio was applied to split the staff between COVID-19 and non-COVID-19 assignments, and further assumptions were made to divide the staff by severity level using data from the MOH detailing the percent of mild, moderate, severe, and critical cases.

However, in both countries it was challenging to collect data regarding how many staff were available by level of COVID-19 severity, since moderate and severe patients were admitted on the same wards and cared for by the same health workers. This meant that the activity standards for the same professional activities required different amounts of time—for example, in Mali a severe patient required 3 h of nursing care in 24 h, whereas a moderate patient required 0.45 h in 24 h.

Challenges with staffing data were further complicated by the confidential nature of health data, particularly involving health workers who had been infected with COVID-19.

### Case data

Challenges in collecting case data were most evident when trying to obtain local data per facility. Case data were available at the national level through daily situation reports; however, local facilities either were unable to prioritize case reporting by severity level, or the information was unavailable due to privacy concerns.

A further issue in obtaining case data related to the challenges presented by undertesting the population. The teams in Mali and Kenya both described that many people in the general population associated testing for COVID-19 with stigma that led many people to stay home rather than present for testing. In Kenya, all travelers into the country and suspected local cases were initially required to pay for mandatory quarantine in designated COVID-19 isolation facilities. Treatment for COVID-19 was free in public facilities while at a cost in private facilities. These approaches inadvertently incentivized many people to hide their symptoms or stay home rather than present for testing. The team in Mali also indicated that many people in the general public would only present for testing as a last resort if they were ill and if their symptoms were not improved by other treatments at home.

### Challenges with updating data

A challenge noted throughout the process was the need to continuously update the tools as the situation on the ground changed rapidly throughout the progression of the pandemic. It was necessary to review assumptions made on a regular basis and revise the tools as needed to reflect the current reality. This challenge was mitigated through frequent communication between both implementing teams to ensure that the modeled data were realistic. For example, a key driver of any infectious disease is how many susceptible contacts each infected individual has an opportunity to spread the infection to. As this was unknown at the start of the pandemic, the Mali team estimated that each individual in densely populated Bamako may be in contact with 20 persons in 1 day. That parameter was then updated using the average number of contacts traced for each positive case, which was included in the government’s daily situation report, to a more realistic 7 and later to 3.5 when new mitigation and suppression measures were enacted. This parameter change was validated by comparing the actual cases reported with the predicted cases from the Adaptt model. In Kenya, closer collaboration with WHO at country level was key in ensuring entry to the national COVID-19 emergency command center to get daily reports/updates.

### Region-specific challenges

Other challenges noted during the phased implementation plans are attributable to regional variations and contexts.

### Kenya

The structure of Kenya’s devolved health sector can create delays in mobilizing a national response. At the beginning of the phased implementation plan, the MOH had several competing priorities and was unable to be as involved. By the later phase of our activity, WHO/AFRO had developed its own African version of the Adaptt model to use with the existing HWFE tool, resulting in ongoing discussion between the MOH and WHO/AFRO on the best tool to use going forward.

### Mali

A challenge noted in the Mali context related to language differences. The Adaptt tool has a language feature, where graphics can be presented in several languages; however, the HWFE is available only in English. The language difference presented a barrier when translating the term for a mechanical ventilator. This initially led to a misunderstanding, where the in-country team was aggregating data for invasive mechanical ventilation, positive pressure ventilation, and all types of oxygen delivery devices, while the remote team was under the assumption that the reported data were for invasive mechanical ventilation only. This misunderstanding was easily resolved by both implementing teams clearly defining their terms, and a lesson learned is to be mindful of language differences when implementing the tools across languages and regions.

### Successes and strengths

A key success of this activity is that the Adaptt and HWFE tools, originally created for European countries, could be repurposed to fit an African context. The tools were well received by the MOH and WHO representatives in Kenya and Mali, and generated realistic data that were used to inform health policy decisions at the regional and national levels. The Kenya and Mali implementing teams focused on adjusting the tools for local resources and context for what was rational and reasonable in their countries, including successfully translating workforce needs for health worker categories and professional activities available in-country.

A strength in the approach used in both countries was being able to use proxy data when the actual daily data requirements were not available for number of new COVID-19 cases. Using estimates from the national COVID-19 response teams for the percentages of patients by level of severity as well as the percentage of cases by region permitted ongoing analysis of the epidemic until actual data from the facilities and regions were available. Similarly, knowing the actual denominator of staff or number of beds and the percentage of staff working with COVID-19 patients or percentage of beds allocated to COVID-19 patients permitted calculation of staff and beds allocated for direct COVID-19 care. It is important for the local team populating these two tools to be in close contact with the national COVID-19 response team to have access to daily data and policy changes being made to the COVID-19 program.

A strength to implementing the tools in Kenya and Mali was access to iHRIS data for the denominator of health workers, which made human resources data collection for COVID-19 easier to obtain. Kenya also benefitted from having completed a WISN assessment in 2013, which facilitated quicker access to workforce activity data for specific cadres and the local understanding of professional activities and activity standards. Even though the MOH in Mali had not completed a WISN, the implementing team was still able to understand the concepts of professional activities and activity standards using the European professional activities and times to obtain the necessary data and incorporate it with assistance from a remote clinical expert.

Another key component in the success of the phased implementation plan was the mirrored roles of the remote and in-country implementing teams, which provided an organized structure to the process and facilitated the appropriate expertise and sharing from each team member. The in-country teams are now equipped to carry the results forward and train others on the use of the tools and interpretation of the results.

It was also noted that obtaining early support from the MOH and involving the country-level WHO and USAID offices and other key stakeholders at the onset of the pilot led to quicker implementation, organized collaboration, and a streamlined process for scaling the results to the rest of the country.

## Conclusion

This phased implementation plan in Mali and Kenya demonstrates that the WISN-type Adaptt and HWFE tools can be readily modified and applied to the local context in non-European countries and support African countries to rapidly estimate the number of health workers and beds needed to respond to the COVID-19 pandemic. The data generated by these modified tools may also be used to provide proxy estimates for needed health supplies—e.g., beds for COVID-19, oxygen, medications, and ventilators.

## Data Availability

The data sets generated and/or analysed during the current study are not publicly available due to limitations on COVID data use by the MoH in Kenya and Mali but are available from the corresponding author on reasonable request.

## Abbreviations

CGTRH: Coast General Teaching and Referral Hospital
COVID-19: Coronavirus disease 2019
HRH: Human resources for health
HWFE: Health workforce estimator
HRIS: Human resources information system
MOH: Ministry of Health
SIR: Susceptible, infected and recovered
USAID: United States Agency for International Development
WHO: World Health Organization
WHO/AFRO: World Health Organization Regional Office for Africa
WISN: Workload indicators of staffing need
